# A Multimodal Imaging Approach for Longitudinal Evaluation of Bladder Tumor Development in an Orthotopic Murine Model

**DOI:** 10.1371/journal.pone.0161284

**Published:** 2016-08-17

**Authors:** Chantal Scheepbouwer, Sandra Meyer, Maroeska J. Burggraaf, Jithin Jose, Carla F. M. Molthoff

**Affiliations:** 1 Department of Radiology & Nuclear Medicine, VU University Medical Center, Amsterdam, The Netherlands; 2 FUJIFILM VisualSonics Inc., Amsterdam, The Netherlands; 3 Department of Medical Microbiology and Infection control, Amsterdam, The Netherlands; AntiCancer Inc., UNITED STATES

## Abstract

Bladder cancer is the fourth most common malignancy amongst men in Western industrialized countries with an initial response rate of 70% for the non-muscle invasive type, and improving therapy efficacy is highly needed. For this, an appropriate, reliable animal model is essential to gain insight into mechanisms of tumor growth for use in response monitoring of (new) agents. Several animal models have been described in previous studies, but so far success has been hampered due to the absence of imaging methods to follow tumor growth non-invasively over time. Recent developments of multimodal imaging methods for use in animal research have substantially strengthened these options of *in vivo* visualization of tumor growth. In the present study, a multimodal imaging approach was addressed to investigate bladder tumor proliferation longitudinally. The complementary abilities of Bioluminescence, High Resolution Ultrasound and Photo-acoustic Imaging permit a better understanding of bladder tumor development. Hybrid imaging modalities allow the integration of individual strengths to enable sensitive and improved quantification and understanding of tumor biology, and ultimately, can aid in the discovery and development of new therapeutics.

## Introduction

Bladder cancer is the fourth most common malignancy amongst men in Western industrialized countries [1]. As the incidence increases with age, the risk of developing bladder cancer is highest in men above 60 years of age with cigarette smoking recognized as the most important risk factor in developing bladder cancer [2]. Even though there is a high variety in the natural course of the disease, approximately 75–85% of bladder tumors are presented as non-muscle invasive limited to the mucosa or submucosa at time of diagnosis [3]. Standard therapy of high-grade non-muscle invasive bladder cancer consists of transurethral resection of the bladder tumor (TURBT), followed by intravesical immunotherapy with *mycobacterium bovis* BCG. This approach is superior to surgical treatment alone with an initial complete response rate of 70% [3–5]. As bladder cancer is a heterogeneous disease, the immune response and tolerance to BCG therapy can vary greatly among patients, so predicting a specific therapeutic outcome has proven to be challenging. Consequently, intensive monitoring is needed, which is reflected in high costs, making bladder cancer one of the most expensive cancer types [6].

Enhancing immunostimulatory properties would be an important step forward in promoting efficacy of BCG therapy for non-muscle invasive bladder cancer [7]. A reliable, simple, and reproducible animal model is essential to gain insight into mechanisms of tumor growth and spread, so these improved therapeutic strategies can be developed for human use [8]. Orthotopic implantation of bladder cancer cells in syngeneic immunocompetent animals has been extensively used in the preclinical testing of altered BCG therapies and research focused on the development of bladder cancer [9, 10]. MB49-luc cells, a carcinogen induced transitional cell carcinoma derived from C57BL/6 mice, are widely used to study these bladder tumors and replicate human urothelial carcinoma cell lines in many molecular and phenotypical responses to BCG *in vitro* [11]. Within this model, several tumor cell implantation procedures exist to establish *in vivo* tumor growth. Still challenging is to longitudinally monitor these bladder tumors, as this requires accurate visualization of tumor growth.

Standard imaging methods in animal studies include radiography (i.e. X-rays), abdominal ultrasound, PET-CT and MRI, however sensitivity for bladder tumor detection is limited [12]. Specifically in radiographic imaging, resolution remains low and limits their utility. PET-CT and MRI greatly improve resolution and can provide 3-dimensional anatomic information [13], but still malignant lesions can be easily missed or misinterpreted.

Over the last years new modalities based on Optical and Ultrasound are emerging for research purposes due to the shortcomings in standard bladder cancer imaging [14, 15]. Fluorescence Imaging (FLI) is one of these new modalities, commonly used to monitor *in vivo* processes with reporters such as green fluorescent protein (GFP), red fluorescent protein (RFP) and near-infrared proteins [16]. Complexity and lower sensitivity due to autofluorescence are the main disadvantages of these reporters [17]. Bioluminescence (BLI) is another commonly used optical imaging modality for both *in vitro* and *in vivo* measurements of molecular and cellular processes. Unlike fluorescence, BLI does not require excitation, thereby avoiding the autofluorescence background signal [18]. BLI is based on the interaction of the luciferase enzymes Firefly luciferase [19], Renilla Luciferase [20] or Gaussia Luciferase [21] with luciferin to catalyze light production in the presence of oxygen and ATP. The luciferase is usually introduced as a reporter gene attached to a regulatory sequence in the DNA. For monitoring bladder tumors, the mouse urothelial carcinoma cell line MB49-luc has the firefly luciferase gene incorporated. This approach is widely accepted and can provide longitudinal quantification of tumor cell proliferation in living animals [22].

High resolution ultrasound (HRUS) has evolved as a rapid, efficient and comparatively inexpensive imaging modality for studying normal development and disease [15]. While clinical ultrasound systems use 3–15 MHz, HRUS emits sound waves ranging from 15–70 MHz [15]. HRUS is a noninvasive technique allowing repeated and frequent measurements in the same animal [23]. Although it allows 3D mapping of the anatomy of the tumor with 30-μm spatial resolution [24], the functional and molecular information of the tumor is limited.

Photoacoustic imaging (PAI) is a hybrid imaging modality that combines optical and ultrasound imaging in real time [25]. In PAI imaging, biological tissue is exposed to a short laser pulse (nano-second) that is absorbed by hemoglobin and other chromophores in tissue causing thermo-elastic expansion, which produces broadband pulses (MHz) of acoustic energy. These pulses propagate to the tissue surface and are detected by an array of ultrasound transducers [26]. Since ultrasound scattering is two to three orders of magnitude weaker than optical scattering in biological tissues [27], PAI can provide a better resolution than optical imaging for depths greater than 1 mm. Furthermore, PAI exploits the different absorption spectra of oxygenated and deoxygenated hemoglobin so relative changes in oxygen saturation can be calculated during simultaneous imaging and determine hypoxic tumor regions in real-time, which can also be directly related to the effect of anti-cancer drugs [28, 29].

In previous studies, BLI, HRUS and PAI have been used as individual techniques for *in vivo* or *ex vivo* bladder tumor imaging [30–32]. The abilities of these different imaging modalities can be exploited by complementary use to increase sensitivity and improve accurate quantification of tumor growth in living animals by providing anatomical, functional and molecular information.

In the present study, we focused on a longitudinal study combining BLI, HRUS and PAI. First, optimal tumor cell inoculation in animals was established Then, the ideal setup for our multimodality imaging was assessed over a time interval of 4 weeks, finalized with histological confirmation. Accordingly, the contribution of each of these non-invasive imaging modalities could be studied for increased sensitivity and accurate quantification of tumor growth in a longitudinal murine orthotopic bladder cancer model, which can result in improved understanding of bladder tumor development.

## Materials and Methods

### Animal Model and Tumor Implantation Procedure

Murine MB49 cells were kindly provided by dr. Y. Luo (Singapore) [8, 11]. Murine MB49-luc cells, made in the lab of dr. T. Wurdinger, VUmc, stably expressing luciferase, were cultured in DMEM (Lonza, Verviers, Belgium) supplemented with 10% fetal calf serum (FCS) and 1% penicillin/streptomycin (Gibco Life Technologies, Grand Island, USA). Cells were authenticated by STR analysis (QIAGEN, Germany) and stability of luciferase expression was regularly checked by limiting dilution methodology (96-well plates) for high expressers (BLI, Xenogen IVIS, see paragraph below).

First, C57Bl/6 mice (female, four-six-week old, Harlan, Horst, NL) were injected subcutaneously (s.c.) [33] in the flank with MB49-luc cells (8E^06^ cells/mL, total of 1E^06^ cells per flank) under 2% isofluran/oxygen anesthetics (0.4 L/min).

Then, C57Bl/6 mice (same origin) were orthotopically implanted with MB49-luc cells under 2% isofluran/oxygen anesthetics (0.4 L/min). First, two methods of tumor cell implantation have been tested: poly L-lysine (PLL) method [34] and local injury method [35] at various cell concentrations (S1 File). Also, several adaptations of the local injury method were implemented by emptying the bladder, flushing with phosphate buffered saline (B. Braun Medical, DE), careful scratching of the bladder wall with a blunted 24-gauge needle creating a local injury and installation of the tumor cells while mice were placed head down on a 10 degrees slope (S1 File and S1 Fig). The established optimal tumor cell concentration of 5E^04^ cells/mL, total of ~3000 MB49-luc cells, were implanted transurethrally using a 24-gauge catheter (Angiocath, Sandy, USA) and left there for 2 h. A urethral clamp prevented leakage and mice received pain medication (0.1-mg/kg s.c. Temgesic, Berkshire, UK), 15 minutes prior to clamp removal. As a control, a sham-operated mouse was investigated using comparable procedures in absence of tumor cells.

After tumor cell inoculation, imaging was performed 2–3 times a week over a time interval of 4 weeks with various techniques. For all imaging procedures, animals were placed on a heating pad to maintain body temperature and abdominal hair was removed (Nair hair removal lotion, Church&Dwight Co., Ewing, USA). For HRUS and PAI images bubble-free ultrasound transmission gel (Parker Laboratories, Warren, USA) was applied at the region of interest. Physiological parameters were maintained at average levels (ECG, range 500 ± 30 beats per minute; respiration rate, 42 ± 6 breaths per minute; body temperature, 28 ± 5°C). Mice were weighed regularly prior and after procedures.

All procedures were performed according to the guidelines of the National Institute of Health, Principles of Laboratory Animal Care, in accordance with the European Community Council Directive 2010/63/EU for laboratory animal care and the Dutch National Law on the use of animals and specifically approved by the Institutional Animal Care and Use Committee of the VU University Medical Center Amsterdam (PET13-04). In the experiments described, at all times human endpoints were applied according to the Dutch Law on animal experimentation and the EU directive on animal experimentation (OECD Environmental Health and Safety Publications, Series on Testing and Assessment, No. 19; Guidance document on the recognition, assessment, and use of clinical signs as humane endpoints for experimental animals used in safety evaluation, 1–19, stating that the human end point should never be spontaneous death of the animal). From the time of arrival all animals have been observed daily on general behavior, appearance, temperature, water and food intake, body weight loss (no more than 15%) visible infections, problems with respect to gastro-intestines, eyes, tremor, breathing, moving, urinary problems, and any other signs of discomfort.

### Bioluminescence Imaging (BLI)

After s.c. injection of 150 μL D-luciferin (Gold Biotechnology, St. Louis, USA) in the neck region, BLI was performed every 6 minutes up to 30 minutes post injection (p.i.) to determine the peak signal and afterwards the optimal time point was applied to all subsequent BLI.

Intensities of BLI signals were monitored by CCD camera using Xenogen IVIS Lumina (Xenogen Corp, Alameda, USA). Regions of interest (ROIs) were drawn around the hot spots as well as for representative background regions for control using Living image software (Perkin Elmer, Massachusetts, USA) and expressed as maximum photons per second.

### High Resolution Ultrasound (HRUS) and Photoacoustic Imaging (PAI)

HRUS and PAI was performed on a Vevo^®^ LAZR Imaging Station (FUIJIFILM VisualSonics Inc., Toronto, ON, Canada). The system features a hybrid ultrasound and photoacoustic transducer (linear array, Central frequency 21 MHz) integrated with a tunable nanosecond pulsed laser (680–970 nm). Photoacoustic oxygenation images were collected at 750 and 850 nm, and the parametric maps of sO2 were calculated based on the two-wavelength approach by using a previously reported algorithm [28]. Three-dimensional (3-D) data sets were collected by linearly translating the transducer with a stepper motor over a region of interest, while capturing two-dimensional (2-D) image (slice thickness of 125 μm) of the 3-D stack. ROIs were drawn around the tumor borders for every slice to assess tumor size in a 3-D dimensional plane and the image analysis software, provided by FUIJIFILM VisualSonics, is used to measure the average oxygen saturation (sO_2_) values within the ROI by calculating the percentage of HbO_2_ in total hemoglobin, using the formula sO_2_ = HbO_2_/(HbO_2_ + Hb) × 100%.

### Histopathology and Immunohistochemistry

At the end of experiments, mice were sacrificed and bladders were fixated *in vivo* for 6 h with 4% neutral buffered formaldehyde via a 24-gauge catheter (Angiocath, Sandy, USA) and processed for paraffin embedding, 4 μm sections were cut and stained with hematoxylin-eosin (HE) for histo-pathological analysis. Additionally, immunohistochemical staining for various markers was performed. Briefly, as a marker for proliferation, 1:200 diluted anti-mouse Ki67 (Pierce Antibodies, Thermo Scientific, Rockford, USA) was used. For analysis of apoptosis, 1:50 diluted anti-mouse/human caspase-3 (Merck Millipore, Darmstadt, Germany) was applied and presence of TRAIL, which may selectively induce apoptosis in cancer cells, was measured with the 1:100 diluted TRAIL antibody (Pierce Antibodies, Thermo Scientific, Rockford, USA). Anti-mouse PECAM-1 (Lifespan Biosciences Inc, Seattle, USA) was used at a dilution of 1:20 for the detection of small vessels in the tumor. CD-3 staining was included with a 1:50 diluted CD-3 antibody (Thermo Scientific, Rockford, USA) as a marker of possible T-cell involvement. For staining of macrophages, sections were incubated with a 1:1000 diluted anti-mouse F4/80 monoclonal antibody (Bioscience, San Diego, USA). Appropriate positive and negative controls were applied throughout.

Two independent observers analyzed all stainings semi-quantitatively as -, ±, +, ++ (negative, weak, moderate and strong, respectively) and the percentage of positive cells was determined for Ki67 and F4/80 stainings.

## Results

### Animal Model

First, tumor take of MB49-luc cells in these syngeneic mice was investigated by injecting 1E10^6^ cells s.c.: tumors developed within 5 days post injection (p.i.) with a tumor take of 100%. Then, optimal parameters were set for the orthotopic approach by testing PLL and local injury methods with varying numbers of MB49-luc cells for maximal interaction between tumor cells and bladder mucosa. An injection of effectively ~3000 cells (concentration of 5E10^4^ cells/ml) was sufficient to develop tumors and animals were sacrificed 30 days post tumor cell implantation. As compared with the PLL method, the mechanical local injury method of tumor cell implantation by direct scraping using a blunted needle allowed more control over the exact tumor location at the cranial pole of the bladder wall, analogous to the clinical pathological process in human.

**Fig 1:**
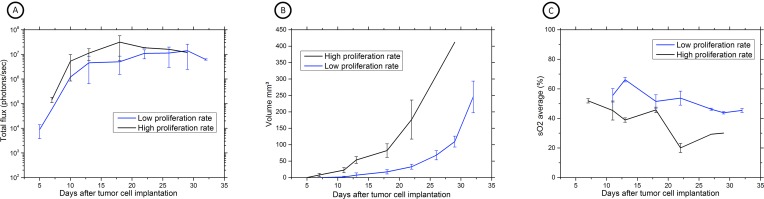
Multimodal imaging assessment during the development of our local injury method of tumor cell implantation. (A) BLI acquisition of MB49-luc tumor-bearing C57Bl/6 mice during local injury method 18 minutes after s.c. luciferin injection. BLI is expressed as the number of photons/sec; the graph is representing the increase in bioluminescence activity over time (n = 7, mean ± SD). Difference in bioluminescence emission is minimal between both groups. (B) HRUS results expressed as tumor volume in mm3 over time after tumor cell implantation (n = 7, mean ± SD). High (n = 2) and low (n = 5) tumor growth kinetics could be observed, thereby distinguishing two growth patterns of tumor formation. (C) Serial PAI measurements showing average sO_2_ values in tumor bearing mice. Similar trends in sO_2_ values can be observed in animals with high (n = 2) and low (n = 5) tumor growth kinetics, though a more pronounced drop in sO_2_ levels was observed in animals with high tumor growth kinetics.

The multimodal imaging assessment was addressed for further development of our local injury method of tumor cell implantation method. As Fig 1 demonstrates, during the development of the model, variations in scratching conditions induced different tumor growth rates, especially seen on HRUS- and PAI-images (Fig 1B and 1C), for BLI, growth variation was less obvious (Fig 1A). With BLI a plateau was reached over time, which might be indicative for the presence of hypoxic regions as confirmed later with PAI.

**Fig 2:**
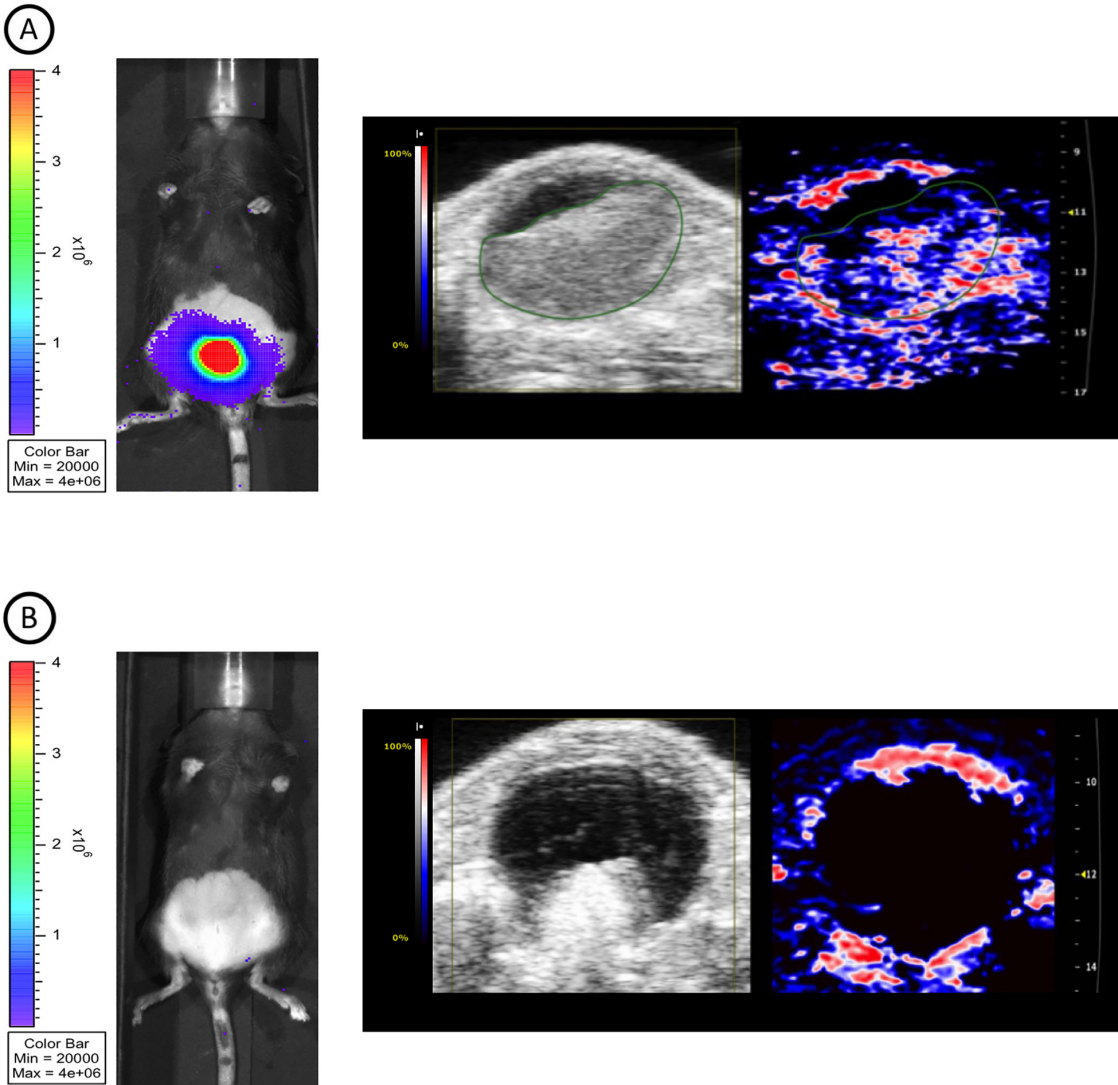
BLI, HRUS and PAI images taken at day 17 post-injection. (A) BLI displays intense luminescence emission (left image), HRUS shows a tumor volume of 102.6 mm^3^(middle image), PAI demonstrates an oxygenated tumor center (right image). Oxygen saturation levels ranging from 0% (blue) to 100% (red). An absence of signal is displayed by black pixels. (B) Discordant results between BLI/PAI and HRUS, demonstrating aberrant growth pattern.

In some mice, no tumor was observed using BLI and PAI (Fig 2B, left and right panel, respectively) while HRUS demonstrated a process inside the bladder lumen. After *ex vivo* investigation of this abnormality it appeared to be a non-tumorous inflammatory process (data not shown).

Minor improvements to the blunted needle, bladder rinsing conditions and performing tumor cell injections under aseptic conditions with mice head down under a 10° slope, resulted in both suspended tumor growth rates and minimal variations in tumor development. This final model was then utilized for the longitudinal multimodality imaging studies.

Applying this optimized method, tumor take measured a reproducible 80% and in all further studies, this local injury method therefore was applied. The sham-operated control did not show tumor growth as expected.

### Multimodality Imaging

#### Bioluminescence Imaging (BLI)

**Fig 3:**
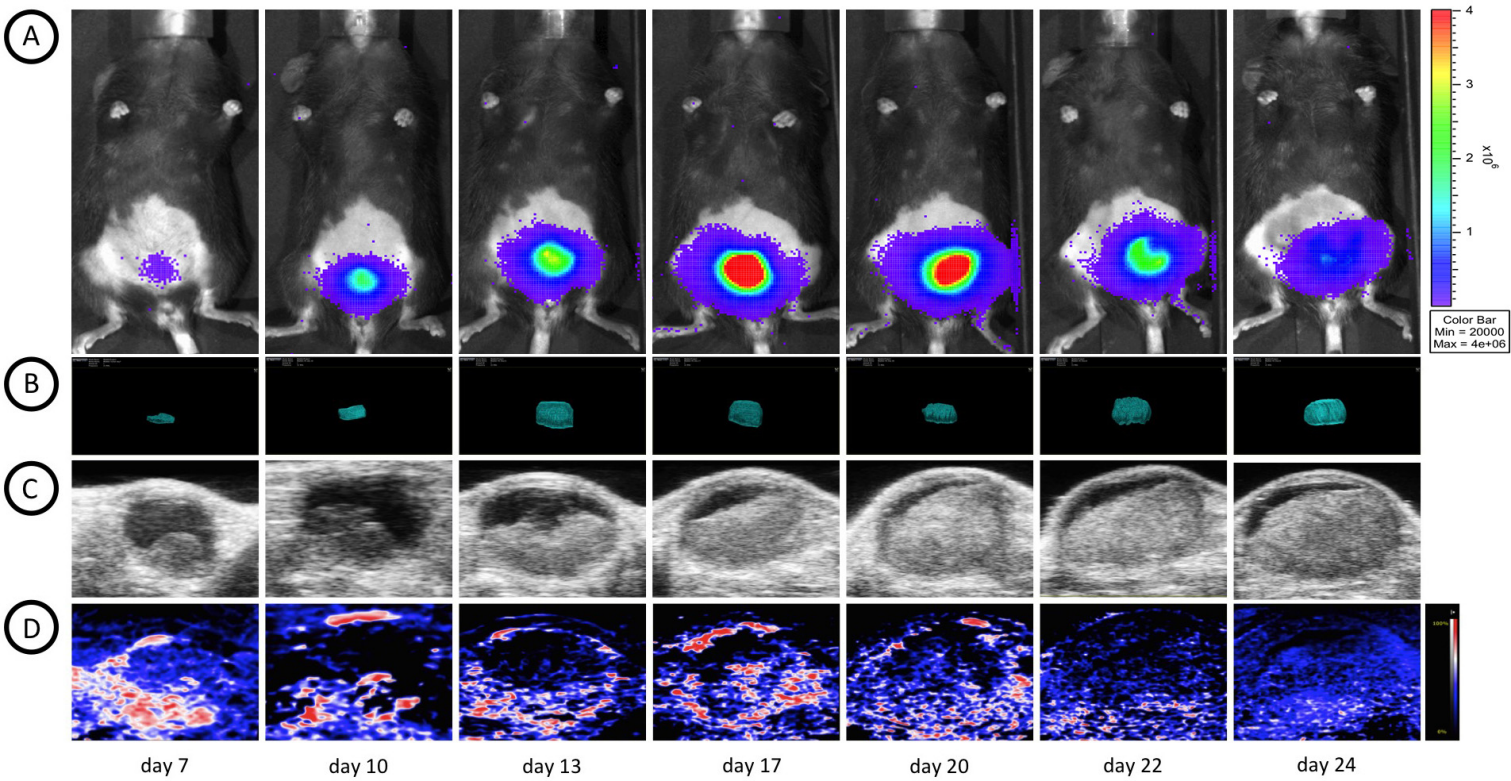
Longitudinal multimodal assessment of bladder tumor growth. All multimodal images were taken at the same days for optimal comparison. (A) bladder tumor growth visualized by serial BLI images of a representative mouse taken 18 minutes after s.c. luciferin injection. An increase in tumor volume can be observed in serial 3D HRUS (B) images and longitudinal 2D sections (C). During the time course of tumor growth a decrease in oxygen saturation (D) from early-stage tumor to later stage tumors was observed.

After installation of the MB49-luc cells in the bladder, tumor growth was monitored non-invasively using BLI. Serial images were taken every 3 minutes to determine the kinetics of luminescence, setting 18 minutes post luciferin injection as the optimal time for imaging. BLI was performed regularly during several sessions over time to analyze tumor cell proliferation. Fig 3A shows representative images of BLI acquisitions over a time interval of 24 days.

**Fig 4:**
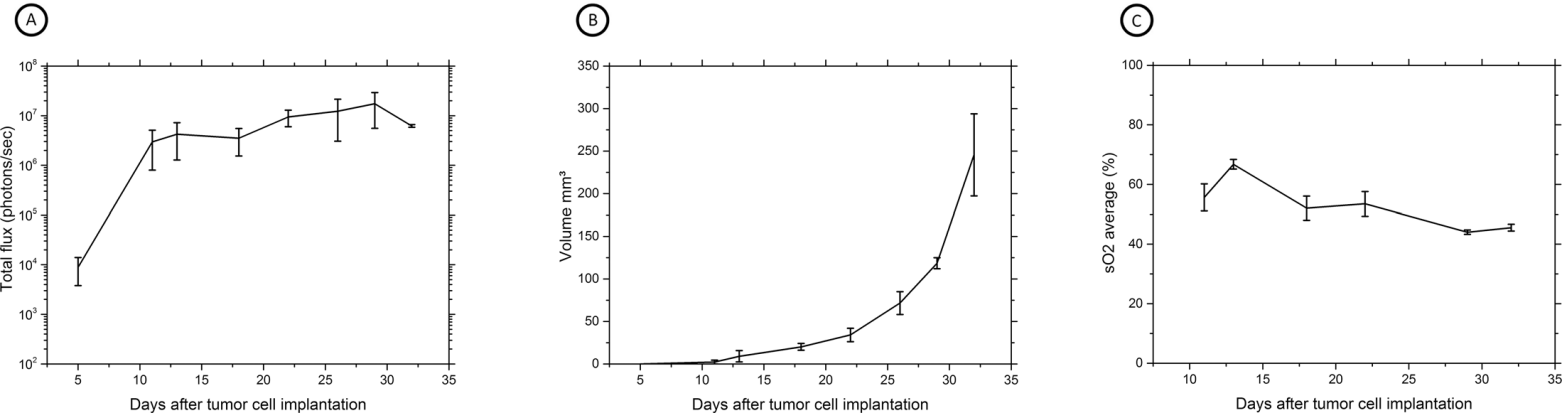
Longitudinal multimodal imaging assessment of *in vivo* bladder tumor growth. (A) BLI acquisition of MB49-luc tumor-bearing C57Bl/6 mice during local injury method 18 minutes after s.c. luciferin injection. BLI is expressed as the number of photons/sec; the graph is representing the increase in bioluminescence activity over time (n = 4, mean ± SD). (B) HRUS results expressed as tumor volume in mm^3^ over time after tumor cell implantation (n = 4, mean ± SD). (C) Serial PAI measurements showing average sO_2_ values in tumor bearing mice (n = 4, mean ± SD).

As shown in Fig 4A, longitudinal BLI measurement was performed with an increase in BLI intensity from the background starting ~5–7 days after tumor cell implantation where after an increase in photon emission occurred, suggesting progressive cancer cell colonization and tumor growth, reaching a plateau between around days 18–32. Mice were then sacrificed. No BLI signal was found in one mouse and this mouse appeared to have no tumor, confirmed by macroscopic and histopathological analysis.

#### High resolution ultrasound (HRUS)

**Fig 5:**
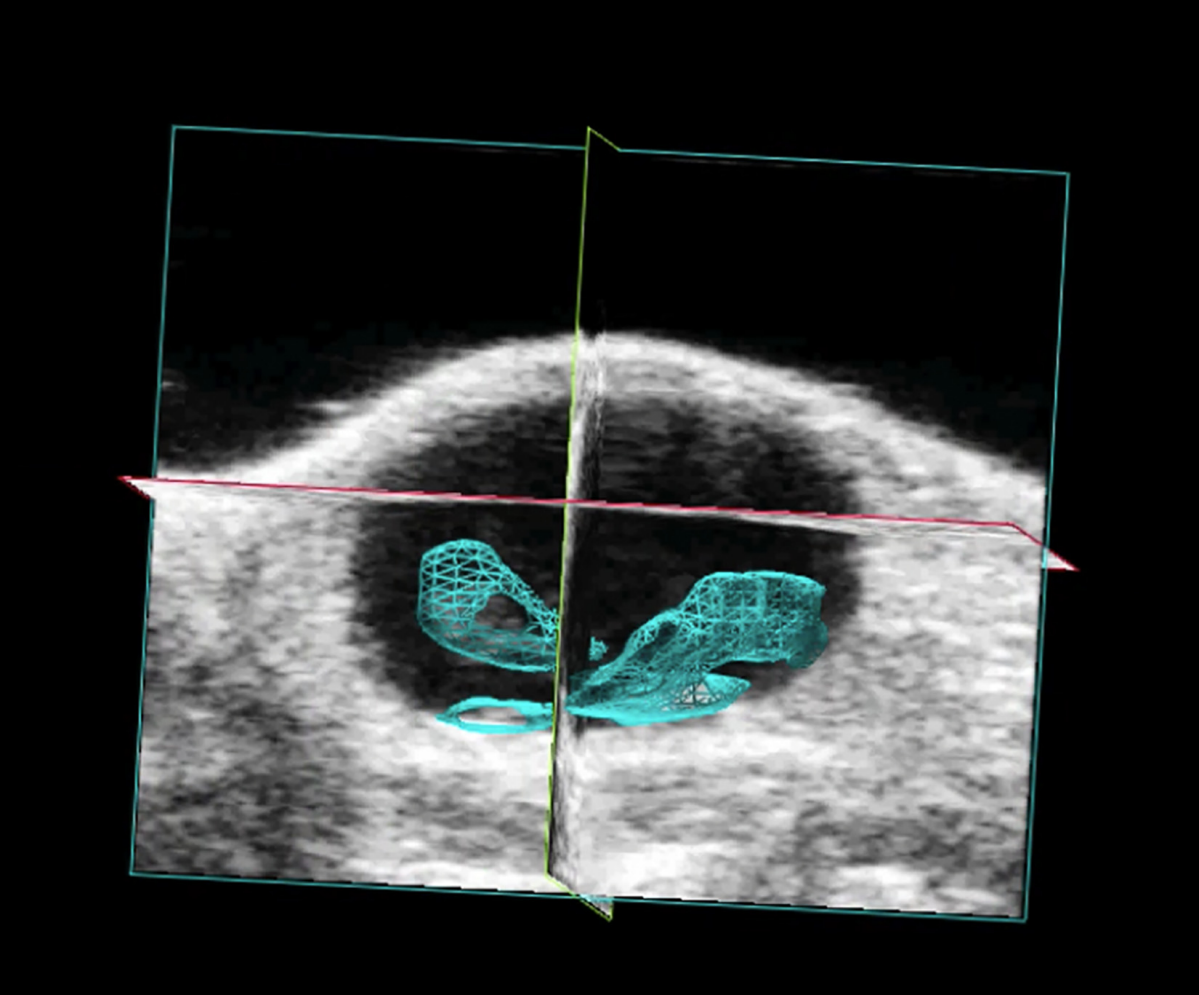
Example of HRUS image. HRUS is able to provide specific anatomical information of bladder tumor development in a 3-dimensional plane. The tumor size was assessed based on the ROIs drawn around the tumor borders for every slice of 125 μm. Tumor is marked in blue.

**Fig 6:**
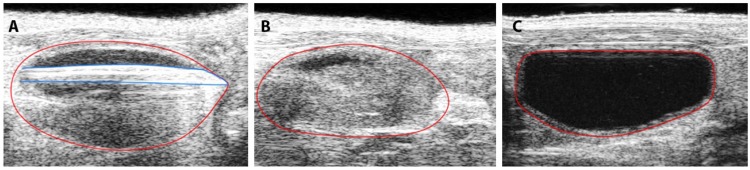
Visualization of the scratching procedure with HRUS. HRUS images were obtained during the optimized local injury method in a sham mouse. Marked in blue and red is the scratching blunted needle and the bladder wall, respectively. (A) scratching of the bladder wall for 1 minute, (B) bladder immediately after the procedure, (C) bladder region after 10 days.

Tumor growth patterns were explored in more detail by exploiting multimodality imaging. All animals were monitored with HRUS at the same timepoints as BLI acquisition (Fig 3B). HRUS confirmed tumor growth in animals as BLI had demonstrated. The smallest detectable tumor was 0.4 mm^3^ at day 11, while the largest detectable tumor was at 308,6 mm^3^ at day 32. Fig 4B shows proportionate tumor growth rates more clearly: tumor formation increased gradually after tumor cell implantation with total volume measurements continued over the remaining time period. Moreover, HRUS could provide specific anatomical characteristics of tumor growth in a 3-dimensional plane (Fig 5). As Fig 5 shows, high-resolution images can inform about irregular tumor growth patterns. Moreover, an HRUS-guided implantation protocol was set-up to address reproducibility and reliability of the model. Fig 6A shows HRUS was able to visualize the scratching procedure in the sham-operated mouse. During our assessment, after 30 minutes, the bladder wall was enlarged and fluid-filled (Fig 6B) without detectable abnormalities 10 days after the procedure (Fig 6C).

#### Photo-acoustic Imaging (PAI)

Photo acoustic oxygen saturation levels of total tumor volumes were monitored over a four-week period to gain insight into the progress of tumor development. In general, early-stage tumors (<10 mm^3^) clearly showed to be highly oxygenated (Fig 3D) and Fig 4C shows an increase in sO2 values in early tumor development. On day 13 peak intensity was observed, followed with saturation untill day 17 and gradual decrease starting at day 22.

#### Histopathology and immunohistochemistry

**Fig 7:**
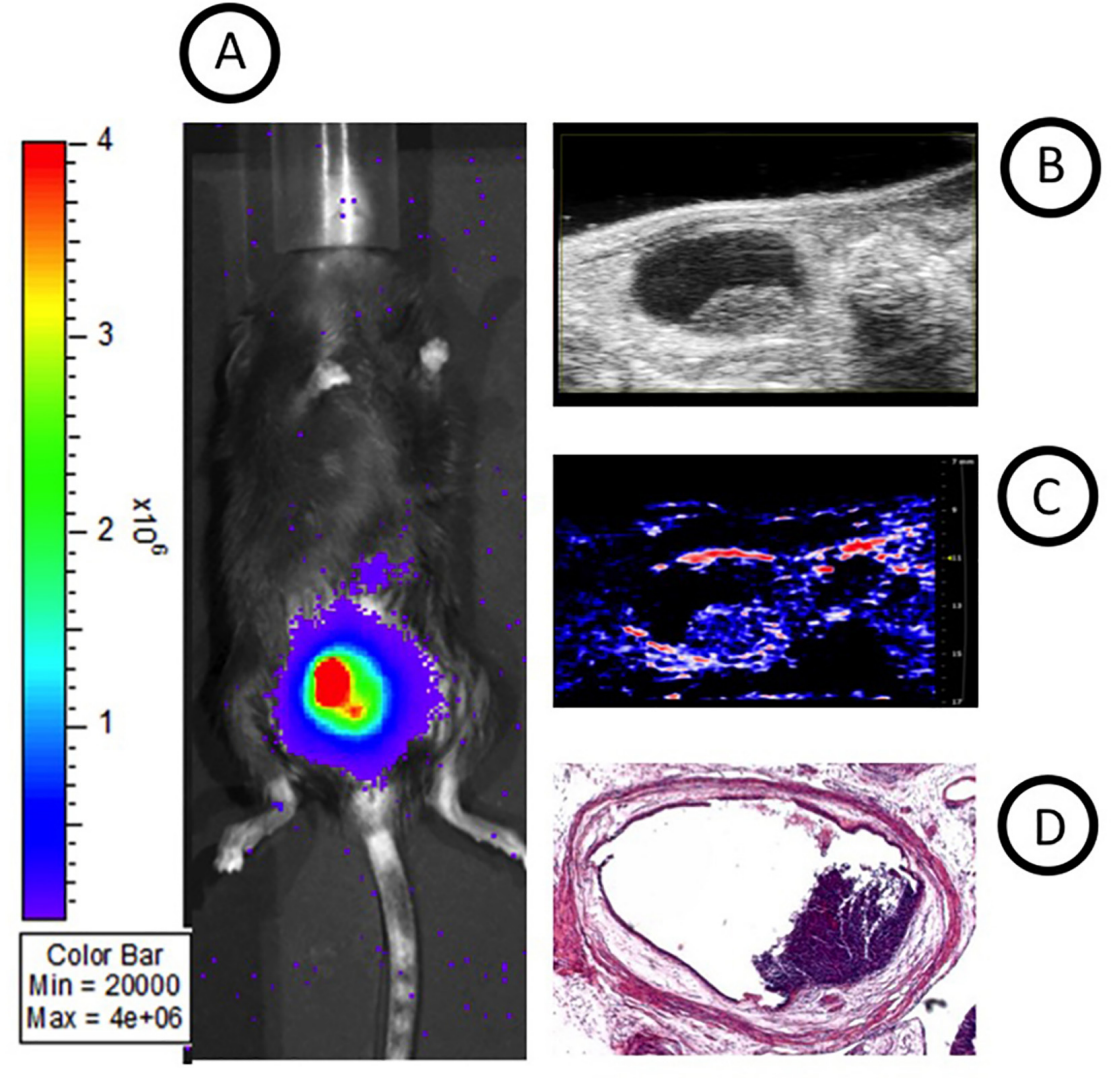
Comparison of BLI, HRUS and PAI images with macroscopic analysis of tumor volumes. (A) BLI image, (B) longitudinal section of the bladder of HRUS image, (C) PAI image and (D) *ex vivo* bladder on sacrificing day for comparison of multimodal images and macroscopic analysis of tumor volumes.

**Fig 8:**
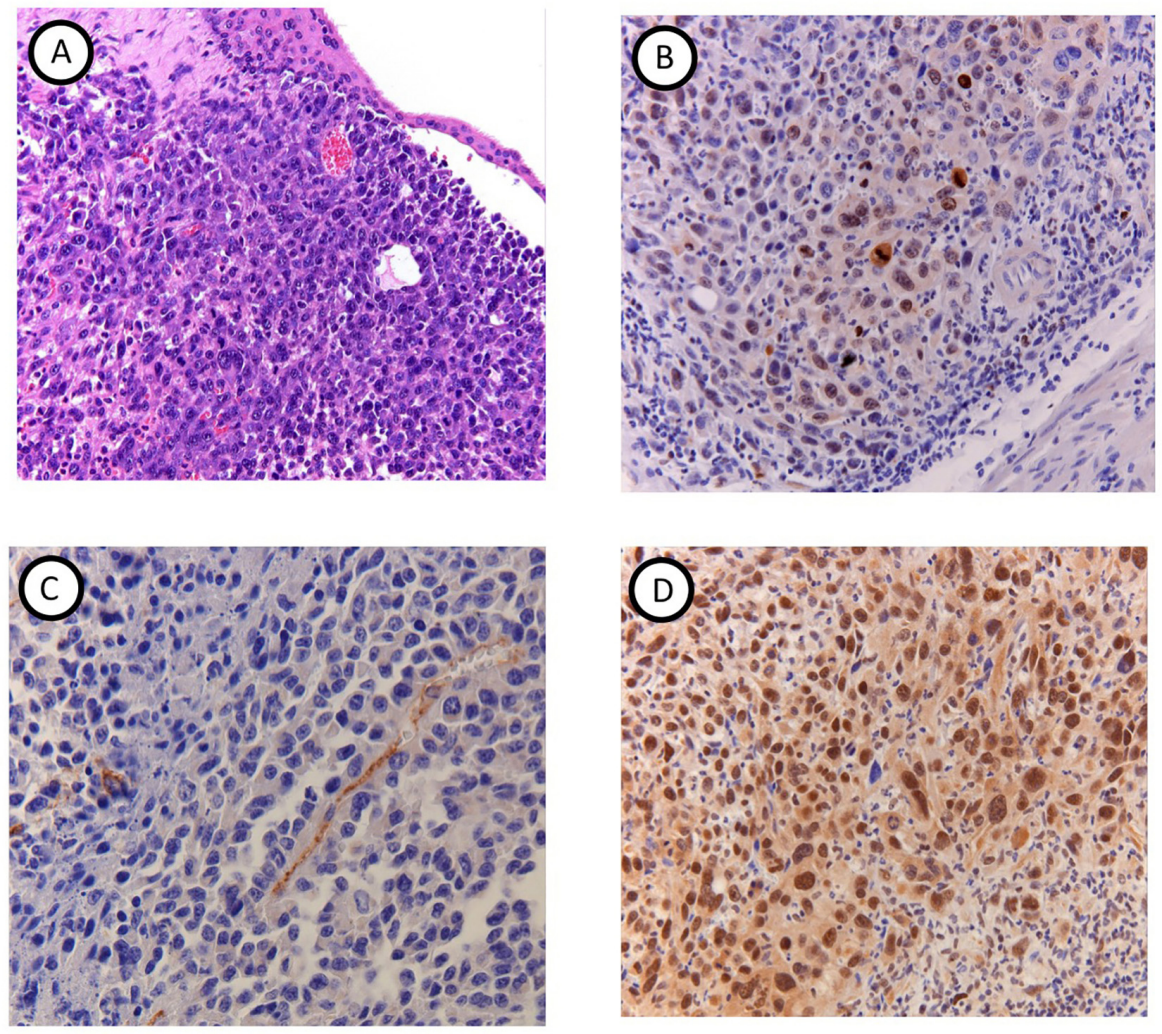
Histopathology and immunohistochemistry. (A) HE images confirm imaging results and show tumor growth extending from the bladder wall in to the bladder lumen. Tumor bearing mice show (B) positive Ki-67 staining, (C) positive PECAM staining as well as (D) high TRAIL expression.

Macroscopic analysis confirmed tumors in the bladder region, consistent with multimodal images (Fig 7A, 7B and 7C**)**. Histological analysis of bladder tumors, created by the local injury method of tumor cell implantation, confirmed tumor cells extending from the bladder wall into the bladder lumen (Fig 7D).

(Immuno)histochemical analysis of tumor tissue revealed areas of necrosis, anaplasia, abnormal mitotic figures and haemorrhage (Fig 8A). All tumors showed positive Ki-67 staining, with moderate to high caspase-3 expression, high TRAIL and PECAM1 expression (Fig 8B–8D**)**. In areas with necrosis, CD3 stainings were found positive and F4/80 stainings were homogenous and showed an average infiltration of 32% (±2%) of macrophages in tumor regions. In non-tumor bearing mice above mentioned markers were negative, with the exception of a strong expression of TRAIL in the bladder wall.

## Discussion and Conclusions

In the present study, we have longitudinally imaged murine orthotopic bladder cancer with a multimodal approach of BLI, HRUS and PAI. Each modality was able to provide complimentary information of tumor growth and kinetics. This approach would be suitable for future preclinical testing of therapeutic agents for improved BCG immune therapy.

Even though multimodality imaging has shown its potential in the assessment of several tumor types, to our knowledge this study is the first report of combining imaging modalities BLI, HRUS and PAI for the purpose of non-invasive longitudinal *in vivo* bladder cancer imaging.

BLI proved to be an easy-to-use, cost-effective imaging modality and it is highly sensitive to assess the *in vivo* tumor growth [31]. The use of HRUS and PAI can provide additional detailed information about the anatomical and functional aspects of tumor development. To date, no PAI studies have been reported for *in vivo* longitudinal evaluation of bladder tumors. Only, two reports studying *ex vivo* bladders were able to show the potential of PAI for bladder cancer research [30, 32] and several reports in ovarian cancer [36, 37].

Our study illustrates that each imaging modality has advantages that can be complementary in characterization of tumor growth. Early-stage tumors (volumes as small as 0,4 mm^3^) can already be detected by HRUS, before BLI showed a luminescence signal. BLI can visualize the tumor cell proliferation non-invasively with high sensitivity and can thus differentiate tumor tissue from infection more easily than HRUS. However, BLI imaging is hampered by strong light scattering in deeper tissue layers, tends to lose the spatial resolution while imaging orthotopic tumors and is dependent on oxygen and ATP. An active cell cycle is needed for the conversion of luciferin, oxygen and ATP into oxy-luciferin, carbon dioxide and ADP by the enzyme luciferase while emitting light. This chemical reaction needs to be kept in mind with respect to BLI.

Also, BLI signals may decrease due to a possible hypoxic core, even though the tumor is still growing as demonstrated by HRUS and PAI. As BLI relies on ATP and O_2_, during longitudinal BLI assessment the bioluminescence signal decreases after reaching a plateau and average O_2_ saturation levels dropped to ~40% as measured by PAI. Another explanation for BLI decrease could be tumor cells switching from aerobic to anaerobic metabolism and advanced tumors outgrowing their blood supply, which leads to poor vasculature in the center of the tumor and thus to necrosis and apoptosis as demonstrated (immuno)histochemically in our study. Decreasing signals could also be caused by loss of luciferase expression. MB49-luc cells kept their original cell profile and no loss of luciferase expression has been seen but cell line authentication and stability of luciferase expression should be checked regularly during experiments.

PAI, a hybrid imaging modality of optical and HRUS has the advantage of obtaining functional and molecular 3D information to gain better understanding of tumor development. In addition, oxygenation measurements, provided by PAI, can be used to monitor the therapeutic effects of new agents.

Orthotopic models of bladder cancer in nude mice have proven to be valuable tools to follow cancer progression into multi-organ metastasis [38, 39]. Furthermore, these xenograft models have shown great value in examining mechanisms of tumor growth and therapeutic response in the treatment of superficial bladder cancer [40, 41]. In the latter studies, fluorescent imaging was applied with GFP-expressing tumor cells to image primary tumors as well as metastases with the advantage of sensitivity and a clear view. A disadvantage of GFP imaging could be the loss of GFP expression over time in some tumor cell lines. However, Hoffman and co-workers demonstrated in their model no loss in the time frame of approximately one month after tumor cell installation [40, 41]. A disadvantage was the invasive nature since an incision had to be made to expose the bladder for each imaging session. Also, the inability to monitor the immune response to intravesical therapy is a major disadvantage [42]. With regard to future preclinical therapy validations, immunocompetence is as crucial as homogeneity and reproducibility of tumor development. Therefore, in line with previous BCG therapeutic studies [9, 10], our ideal setup was the use of a syngeneic model using the local injury method of tumor cell implantation [42]. The final mechanical local injury method of creating subtle injury to the mucosa of the urinary bladder contributed to reproducible orthotopic bladder tumor development corresponding to the clinical situation. Moreover, we have demonstrated that the multimodal-imaging approach provides complementary information of tumor growth and kinetics *in vivo*. Our line of reasoning is that multimodal imaging will further improve the information retrieved from the mouse model when used for clinical testing of improved BCG constructs.

The individual modalities also have some limitations that can be overcome using a multimodal imaging approach as shown in this study. Analysis of the results of the individual imaging modalities might raise the risk of misinterpreting results. For instance, HRUS may display growth patterns resembling tumor growth while BLI clearly shows an absence of proliferating tumor cells. At the other hand, during excessive tumor growth BLI fails to accurately quantify tumor growth while HRUS can still accurately visualize and measure tumor development. Irrelevant to HRUS monitoring, but critical for BLI and PAI is the development of skin pigmentation in C57Bl/6 mice during maturation, which influences results and require further investigation in future experiments.

All together, the combination of BLI, HRUS and PAI provides an attractive platform for monitoring bladder tumors longitudinally, while in literature BLI, HRUS and PAI are commonly used as independent imaging modalities. By providing complementary information on structural and functional aspects of tumor development, multimodality imaging appears to be of essence to the analysis of these tumors. These properties can be further utilized during future preclinical therapy testing. Moreover, we provided a set-up for HRUS guided implantation procedure, which allows more control during the scratching procedure. Accordingly, in future we will investigate the possibility of integrating a CCD camera into the current imaging setup of HRUS and PAI and thus combining BLI, HRUS and PAI in to one imaging modality. This approach will allow the integration of individual strengths of each modality to enable sensitive and improved quantification of tumor biology.

## Supporting Information

S1 Fig Experimental procedure. (left) Enlargement of a blunted 24-gauge needle for creating a local injury of orthotopic implantation with MB49-luc cells in C57Bl/6 mice. (right) Experimental setup with graphical representation of a 24-gauge catheter insertion into the bladder in blue.
(TIF)

S1 File Experimental procedure. (DOCX)
