# Supplementary figures and images for: A Multimodal Imaging Approach for Longitudinal Evaluation of Bladder Tumor Development in an Orthotopic Murine Model
Source: PLoS One. 2016 Aug 17;11(8):e0161284. doi: 10.1371/journal.pone.0161284 (PMC4988778; doi:10.1371/journal.pone.0161284)

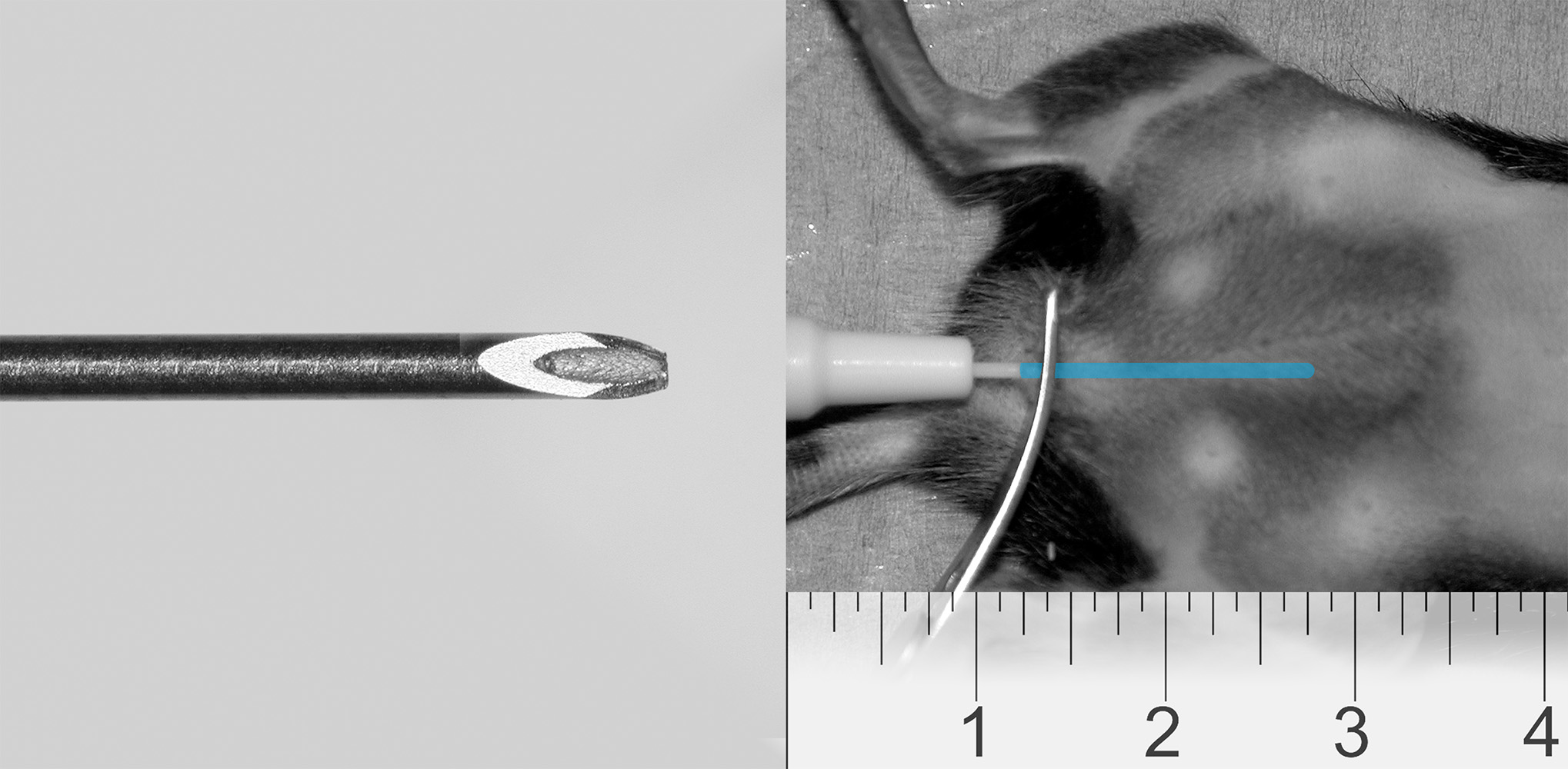

Supplement: S1 Fig — (left) Enlargement of a blunted 24-gauge needle for creating a local injury of orthotopic implantation with MB49-luc cells in C57Bl/6 mice. (right) Experimental setup with graphical representation of a 24-gauge catheter insertion into the bladder in blue. (TIF) [file pone.0161284.s001.tif]
