# Supplementary material for: A Multimodal Imaging Approach for Longitudinal Evaluation of Bladder Tumor Development in an Orthotopic Murine Model
Source: PLoS One. 2016 Aug 17;11(8):e0161284. doi: 10.1371/journal.pone.0161284 (PMC4988778; doi:10.1371/journal.pone.0161284)
Supplement: S1 File — (DOCX) [file pone.0161284.s002.docx]

**S1 File. Experimental procedure.**

C57Bl/6 mice (female, four-week old) were orthotopically implanted with MB49-luc cells under 2% isofluran/oxygen anesthetics (0.4 L/min). First, the orthotopic approach was tested using the poly L-lysine method (PLL) and varying numbers of MB49-luc cells to establish the desired tumor cell concentration. After emptying the bladder and flushing it with 0.9 % sterile NaCl, a 24-gauge catheter (Angiocath, Sandy, USA) and thereafter 100 µl PLL was inserted into the bladder and left inside for 20 minutes. Then, the PLL was squeezed out from the bladder and MB49-luc cells at various concentrations were implanted transurethrally using a 24-gauge catheter (Angiocath, Sandy, USA) and left there for 2 h. A urethral clamp prevented leakage. An injection of effectively ~3000 cells (concentration of 5.10^4^ cells/ml) was sufficient to develop tumors and animals were sacrificed 30 days post tumor cell implantation. Unfortunately, the tumor’s precise location of origin cannot be controlled using the PLL method. To address this issue, the local injury method was used to gain more control over the exact tumor location within the bladder wall.

Therefore, second, the local injury method was applied. When using the local injury method, careful scratching of the bladder wall after insertion of a blunted 24-gauge needle created a local injury (S1 fig). Thereafter, varying cell numbers were implanted transurethrally using a 24-gauge catheter (Angiocath, Sandy, USA) and left there for 2 h. A urethral clamp prevented leakage and mice received pain medication (0.1-mg/kg s.c. Temgesic, Berkshire, UK) 15 minutes prior to clamp removal. Again, an injection of effectively ~3000 cells (concentration of 5.10^4^ cells/ml) appeared sufficient with respect to the preferred time window of tumor growth. Moreover, the local injury method allowed for tumor growth at the cranial pole of the bladder wall.

After establishment of the preferred implantation model regarding tumor cell concentration (5E^04^ cells/mL, total of ~3000 cells), further optimization included flushing the bladder with phosphate buffered saline (Braun Medical, DE) instead of sodium chloride 0.9%. Also, during installation of the tumor cells, mice were placed head down on a 10 degrees slope, rather than horizontally. All procedures were performed under aseptic surgical conditions.
